# Home-based pre-exposure prophylaxis (PrEP) services for gay and bisexual men: An opportunity to address barriers to PrEP uptake and persistence

**DOI:** 10.1371/journal.pone.0189794

**Published:** 2017-12-27

**Authors:** Steven A. John, H. Jonathon Rendina, Christian Grov, Jeffrey T. Parsons

**Affiliations:** 1 Center for HIV/AIDS Educational Studies & Training, Hunter College of the City University of New York (CUNY), New York, NY, United States of America; 2 Department of Psychology, Hunter College of the City University of New York (CUNY), New York, NY, United States of America; 3 Doctoral Program in Health Psychology and Clinical Science, The Graduate Center of CUNY, New York, NY, United States of America; 4 CUNY Graduate School of Public Health and Health Policy, New York, NY, United States of America; University of New South Wales, AUSTRALIA

## Abstract

Gay, bisexual, and other men who have sex with men (GBM) are disproportionately affected by the HIV epidemic. Despite the promise of pre-exposure prophylaxis (PrEP) in reducing HIV transmission risk, barriers for uptake and persistence exist. We sought to identify whether GBM in a nationwide cohort who have not yet initiated PrEP (*n =* 906) would prefer to get PrEP-related care from a primary care provider (PCP) compared to a specialist clinic or provider. We then sought to identify their level of interest and factors associated with preference for using home-based PrEP services (i.e., HB-PrEP), defined to participants as conducting HIV/STI self-testing from home with PrEP prescription mailing after an initial in-person clinic visit. We examined the associations of demographics, sexual HIV transmission risk, concern about frequent medical checkups associated with PrEP, health care access, and PrEP intentions with preferences for healthcare provider type and HB-PrEP. Concern about frequent medical checkups were associated with preferring a PCP for PrEP-related care, but men who perceived a barrier to bringing up the topic of PrEP with a doctor preferred a specialist clinic or provider more than a PCP. HB-PrEP was more appealing for younger men and those engaged in sexual HIV transmission risk, suggesting HB-PrEP could help reach GBM most vulnerable to HIV and in need of PrEP. HB-PrEP expansion has potential to increase PrEP uptake and persistence among GBM, particularly for men with barriers to clinic-based care and higher intentions to initiate PrEP. Clinical guidelines regarding HB-PrEP are needed to expand its use.

## Introduction

Over 1.2 million people are living with HIV in the United States (US), and over 40,000 new cases of HIV are diagnosed each year nationally [1]. Gay, bisexual, and other men who have sex with men (GBM) are disproportionately affected by HIV, representing 67% of all incident infections of HIV and 84% of new infections among males in the US during 2015 [1]. New biomedical mechanisms of HIV prevention are emerging, including the use of pre-exposure prophylaxis (PrEP). PrEP is currently FDA approved as a once-daily pill (emtricitabine/tenofovir disoproxil fumarate) to reduce HIV acquisition risk [2], which has shown ability to reduce HIV seroconversion by up to 92% in clinical trials [3]. The Centers for Disease Control and Prevention (CDC) estimates that as many as one in four GBM would benefit from the HIV protection PrEP offers [4], and some researchers have found that this proportion may even be as high as two-thirds [5].

Uptake of PrEP among GBM has been slow despite increasing awareness of PrEP [6–8]. One US national study of GBM found that 64% met CDC-criteria for PrEP use [5]; however, only an estimated 1% of the estimated 4.5 million US national GBM who may be good candidates for PrEP are currently prescribed it [5, 9, 10]. The Motivational PrEP Cascade is a continuum used to understand important milestones for PrEP uptake and persistence [5]. In this cascade, men willing and intending to initiate PrEP must know of and speak to a medical provider to obtain a PrEP prescription, and those who initiate PrEP require quarterly follow-up appointments for HIV testing as part of PrEP persistence. Even among those GBM who know of a potential PrEP provider and are intending to initiate PrEP, over 47% had not spoken to a medical provider and obtained a PrEP prescription [5].

Research is currently limited as to where GBM want to receive their PrEP-related care. Discomfort in talking to a doctor about PrEP is documented as one potential barrier to initiating PrEP [11]. Among GBM, awareness of PrEP has been associated with having a healthcare provider who is aware that they have sex with other men (i.e., “out”), potentially facilitating culturally competent HIV prevention [12]; however, men who had not discussed their same-sex sexual behavior with a healthcare provider were less willing to use PrEP [13]. Discussing PrEP can be difficult for many GBM because of sexual minority stigma [14]. For Black GBM, race-based distrust in the medical system has been associated with lower willingness to use PrEP [13], highlighting additional barriers that may be faced by GBM of color–as both racial/ethnic and sexual minorities–in obtaining a PrEP prescription. Researchers have previously indicated the importance of structural factors on awareness and intentions to initiate PrEP, but no known prior research has explicitly asked GBM where and with whom they would prefer to receive their PrEP related-care.

PrEP use requires maintaining adherence to once-daily dosing and frequent HIV testing, which create barriers for both PrEP uptake and persistence. PrEP persistence can be defined as maintaining all aspects of PrEP care, including adequate adherence to medications and obtaining HIV and sexually transmitted infection (STI) testing every three months, as recommended [15]. Concerns about needing to maintain daily adherence to PrEP have been identified as a barrier to its use among GBM [16, 17], but research is currently underway to study alternative mechanisms for dosing, such as on-demand and long-acting injectable PrEP, among others [18, 19]. Nonetheless, many GBM who have initiated PrEP have reported high adherence; nearly 98% of men who started PrEP in a US national sample of GBM reported adequate dosing for optimal protection of four or more doses per week [5], and 78–86% of PrEP users had tenofovir diphosphate drug levels in the highly protective range in demonstration projects with biologically confirmed testing [20, 21]. Alternative methods of PrEP dosing could reduce barriers to PrEP uptake for some men who have concerns about once-daily medication adherence [22]. However, research is currently limited on reducing barriers to PrEP uptake associated with quarterly HIV testing requirements of PrEP use; committing to routine medical check-up requirements has been identified as a large barrier to PrEP uptake among GBM [16, 17]. Despite high adherence to PrEP in a US national sample of GBM, only 72% of the men on PrEP had returned for quarterly HIV testing [5]. Interventions that address PrEP persistence issues specifically related to meeting follow-up clinic visit schedules are needed to expand uptake and maintenance of PrEP use.

Advancements in at-home, self-administered HIV and bacterial STI testing make it feasible to meet quarterly HIV/STI testing requirements for PrEP use without the necessity of a clinical visit for each follow-up appointment. HIV self-testing is a highly acceptable method of HIV testing for GBM [23–27]. Mechanisms of self-collecting oral fluid with samples returned by mail (e.g., Orasure HIV-1 Oral Fluid Collection Device; Orasure Technologies) [28] can provide laboratory-based testing services, which has demonstrated antibody reactivity within a window period less than 30 days after exposure [29]. Moreover, the use of dried blood spot testing could be integrated as a method of adherence management, which can detect drug levels by testing fingerpick droplets of blood collected on filter paper and transported at ambient temperature with desiccant [30, 31]. At-home bacterial STI testing–both urethral and rectal–has also been identified as a feasible testing strategy for GBM [32], and at-home bacterial STI testing has demonstrated the ability to be a cost-effective alternative to clinic-based testing [33]. Each of these biomedical advancements could be used as tools for expansion of PrEP persistence management, particularly useful for GBM who want to initiate PrEP but have barriers to the routine follow-up requirements for facility-based HIV/STI testing.

The purpose of this paper was to study the preferences of GBM who have not yet initiated PrEP (i.e., PrEP-naïve GBM) to fill critical gaps in the literature. First, we sought to identify whether PrEP-naïve GBM would prefer to get PrEP-related care from a primary care provider (PCP) compared to a specialist clinic or provider. We hypothesized men who had health insurance and were “out” to their PCP would prefer to receive PrEP-related care from their PCP and, conversely, that men without access to an LGBT-friendly provider would prefer to receive PrEP-related care from someone other than their PCP. Given the frequent nature of medical visits for PrEP persistence, we hypothesized men with more concern about frequent medical check-ups would prefer to receive their care from a PCP. Alternatively, we hypothesized men who had more concern about bringing up the topic of PrEP with a doctor might prefer a specialist because this provider might not be their regular physician they need to see regularly for non-PrEP health care. Finally, we hypothesized fewer non-White GBM would prefer a PCP because of the unique stigma barriers for these men identified in prior research [11, 13, 14], but we made no other hypotheses about demographic factors and sexual behavior on healthcare provider preferences.

Second, we sought to identify the level of interest and factors associated with preference for using home-based PrEP services (i.e., HB-PrEP), which we described as using self-administered HIV/STI testing from home with prescription mailing after an initial clinical visit for PrEP prescription. We hypothesized that GBM with less access to applicable health care, including access to a PCP or LGBT-friendly provider located nearby, would be more likely to be interested in HB-PrEP. We also anticipated men with more concern about frequent medical check-ups and more concern about discussing PrEP with a doctor to prefer HB-PrEP. We had no hypotheses for HB-PrEP preference by demographics or sexual behavior. We present this paper with both outcomes together because GBM who might be interested in HB-PrEP would still need to obtain a prescription for PrEP, which would require seeing a primary care or specialist provider.

## Methods

Data used for these analyses were collected as part of the *One Thousand Strong* study [34], a national cohort of 1,071 GBM in the US. *One Thousand Strong* is a longitudinal cohort of HIV-negative (at baseline) GBM prospectively followed over time. Recruitment methods used targeted sampling to ensure adequate representation of same-sex households based on age, race/ethnicity, and US geography. Enrolled men had a confirmed HIV-negative test result at baseline, and cross-sectional data from the 12-month follow-up were used for this analysis. During the 12-month follow-up assessment in the second half of 2015, participants were asked about their preferences for receiving PrEP-related care and interest in using home-based PrEP services (i.e., HB-PrEP). Recruitment and enrollment details are described elsewhere [5, 17, 32, 34–36]. All study procedures were approved by the Institutional Review Board of the City University of New York.

Of the 1,071 GBM enrolled at baseline, 1,017 (95.0%) completed the 12-month follow-up assessment. Another eight men were excluded (*n* = 1,009) because they did not complete the PrEP questionnaire supplement. As the goal of these analyses was to identify mechanisms to increase PrEP uptake, men had to self-report HIV-negative (*n* = 1,005) at the time of the 12-month assessment and not be currently or previously prescribed PrEP. This resulted in a final analytic sample of 906 HIV-negative, PrEP-naïve GBM.

### Measures

#### Demographics

Demographic data collected from participants included age, race/ethnicity, educational attainment, employment status, income, geographic region of residence determined from postal codes, and relationship status.

#### Sexual HIV transmission risk

Participants were asked about their main partners’ HIV-status, and we also asked participants about behavior indicative of sexual HIV transmission risk. Men were coded as engaging in sexual HIV transmission risk if they had any condomless anal sex with an HIV-positive or unknown partner and/or any casual partner in the past three months.

#### Structural barriers to PrEP-related care

All men enrolled in this study were tested for HIV at baseline and again at the 12-month follow-up; therefore, we asked whether men were tested for HIV only within the last six months (excluding the test performed as part of the 12-month follow-up). Men were also asked if they had health insurance, and we created a categorized variable to measure primary care provider (PCP) access. Men were categorized as: 1) not having a PCP, 2) having a PCP, but not reporting being “out” to their PCP, and 3) having a PCP who is aware that the participant has sex with men (i.e., “out”). Finally, men were asked whether they had access to an LGBT-friendly healthcare provider that was located within 30 minutes travel time.

#### PrEP-intentions and PrEP-related care concerns

PrEP intentions were measured by a single question [5, 17, 35, 36]: “Do you plan to begin PrEP?” Response categories ranged from 1 (*no*, *I definitely will* *not* *begin taking PrEP*) to 5 (y*es*, *I will definitely begin taking PrEP*). PrEP-related concerns [16, 37] most relevant to structural barriers to care were used for analysis. Specifically, participants were asked: “[When thinking about whether to take PrEP, how concerned are you about]… Having to return for medical check-ups every three months while I am on PrEP?” and similarly “… Having to talk to a doctor about your sex life?” Response categories ranged from 1 (*not at all concerned*) to 4 (*very concerned*).

#### Preferences for receiving PrEP-related care from a PCP

Participants were asked where they would prefer to receive their PrEP-related care with the following question: “Suppose that you were interested in getting a new prescription for PrEP–where would you feel most comfortable receiving your PrEP-related medical care and prescriptions?” Men who selected *my primary care provider (my regular doctor)* were coded as preferring to receive PrEP-related care from a PCP, whereas men who answered any of the specialty clinic choices [*A clinic specializing in*: *1) HIV-related care (e*.*g*., *an HIV clinic)*, *2) Sexual health (e*.*g*., *a Planned Parenthood*, *an STD clinic)*, *3) LGBT health care*] were coded as preferring specialist clinics and providers. Men who selected *other* (i.e., the fifth answer choice; *n =* 17) were recoded as preferring specialist clinics and providers, unless their open-text response indicated preference for a PCP or “all of the above” resulting in recoding as preferring a PCP.

#### Preference for using HB-PrEP

We asked participants about their preference about using home-based PrEP services with the following question: “Suppose there was a service where, after one in-person medical visit, you could then conduct self-administered, HIV/STI testing from home with a new prescription mailed to you every three months instead of visiting your medical provider. Which of the following would you prefer?” Response categories were: *1) I would prefer to use this service*, *2) I would prefer to continue visiting a medical provider every three months*, and *3) I have no preference*. Participants were coded as preferring HB-PrEP if they selected the first response, whereas men who preferred continuing to visit their medical provider or had no preference were coded as not preferring HB-PrEP.

### Statistical analyses

Bivariate associations with indications of PrEP-related care preference outcomes were conducted using chi-squared comparisons and logistic regression. All variables that were hypothesized as potential confounders were included in the multivariable models regardless of statistical significance in bivariate analyses, as bivariate analyses only demonstrate the impact of the variable on the outcome and not on its potential confounding effect (i.e., on the association between a predictor and outcome). For our fully-adjusted regression models, we used multivariable binary logistic regression calculated separately for: 1) preference receiving PrEP-related care from a PCP, and 2) preference using HB-PrEP.

## Results

Of the 906 HIV-negative, PrEP-naïve men in this study, most were White (72.1%), had a Bachelor’s degree or higher education (58.7%), and were employed at least part-time (85.7%). About half (47.5%) made more than $50,000 in annual income, and average age was 41.9 years old. Roughly half (53.3%) were in a relationship, and 54 (6.0%) had an HIV-positive main partner. One-third of the sample had engaged in sexual HIV transmission risk in the past three months, and 41.7% were tested for HIV in the past 6 months between our survey and testing assessments. Most (90.4%) had health insurance, but a quarter (25.3%) of men did not have a PCP. Of those with a PCP, 78.6% of them reported that their PCP knew they had sex with men. Thirty-nine percent of the sample did not report having access to an LGBT-friendly provider within 30 minutes of travel. On average, intentions to initiate PrEP were modest among the men in this sample. The average response was just below the threshold of “I might take it,” and men reported a moderate amount of concerns about PrEP. On average, men were “a little concerned” about medical check-ups every three months and bringing up the topic of PrEP with a doctor. See *Table 1*for a full description of the sample.

**Table 1:** Demographics, sexual risk behavior characteristics, health care access, and PrEP-related factors and their associations with 1) preference to receive PrEP-related care from a primary care provider and 2) interest in using home-based PrEP services (*n* = 906).

			Prefer Receiving PrEP-Related Care from a PCP^1^ (Ref: prefer specialist clinics and providers)			Prefer using Home-Based PrEP Services (Ref: not preferred)		
Categorical Variables	*n*	*Column %*	*n*	*Row %*	χ^2^	*n*	*Row %*	χ^2^
Race/Ethnicity					12.9**			1.6
Black	70	7.7	37	52.9		50	71.4	
Latino	109	12.0	50	45.9		77	70.6	
White	653	72.1	395	60.5		470	72.0	
Other/Multiracial	74	8.2	34	46.0		58	78.4	
Education					0.7			1.2
Less than Bachelor’s degree	374	41.3	207	55.4		263	70.3	
Bachelor’s degree or more	532	58.7	309	58.1		392	73.7	
Employment					0.0			6.4*
Unemployed	130	14.4	74	56.9		82	63.1	
Employed (part-time or full-time)	776	85.7	442	57.0		573	73.8	
Income					21.2***			0.3
Less than $20k per year	131	14.5	63	48.1		97	74.1	
$20k to $49k per year	345	38.1	174	50.4		250	72.5	
$50k or more per year	430	47.5	279	64.9		308	71.6	
Geographic Region					3.5			4.8
Northeast	172	19.0	106	61.6		115	66.9	
Midwest	168	18.5	101	60.1		130	77.4	
South	318	35.1	174	54.7		229	72.0	
West	248	27.4	135	54.4		181	73.0	
Relationship Status					5.2*			1.9
Single	423	46.7	224	53.0		315	74.5	
In relationship	483	53.3	292	60.5		340	70.4	
Main Partner HIV-Status					2.2			2.5
HIV-negative, unknown, or no main partner	852	94.0	480	56.3		621	72.9	
HIV-positive	54	6.0	36	66.7		34	63.0	
Engaged in Sexual HIV Transmission Risk^2^					0.7			11.6***
No	604	66.7	350	58.0		415	68.7	
Yes	302	33.3	166	55.0		240	79.5	
Recent HIV Testing (Within last 6 months)					0.0			0.0
No	528	58.3	300	56.8		383	72.5	
Yes	378	41.7	216	57.1		272	72.0	
Has Health Insurance Status					12.5***			0.1
No	87	9.6	34	39.1		64	73.6	
Yes	819	90.4	482	58.9		591	72.2	
Primary Care Provider (PCP) Access					157.4***			21.0***
No PCP	229	25.3	72	31.4		188	82.1	
Not "out" to PCP	145	16.0	49	33.8		112	77.2	
"Out" to PCP	532	58.7	395	74.3		355	66.7	
An LGBT-Friendly Healthcare Provider is Located Within 30 Minutes Travel Time					28.6***			10.9***
No or don't know	349	38.5	160	45.9		274	78.5	
Yes	557	61.5	356	63.9		381	68.4	
Continuous Variables	*M*	*SD*	*OR*	*SE*		*OR*	*SE*	
Age	41.9	13.9	1.02***	0.01		0.98***	0.01	
PrEP Intentions (Range: 1–5)	2.6	1.0	0.90	0.06		1.51***	0.12	
Concern about Medical Checkups Every 3 Months (Range: 1–4)	2.1	1.1	0.93	0.06		1.43***	0.11	
Concern about Bringing up the Topic of PrEP with Doctor (Range: 1–4)	1.8	1.0	0.46***	0.04		1.58***	0.14	

* *p* < 0.05.

** *p* < 0.01.

*** *p* < 0.001.

^1^ PCP = primary care provider.

^2^ Any condomless anal sex with an HIV-positive or unknown partner and/or any casual partner in past 3 months.

Percentages may not add up to 100 because of rounding.

### Preference receiving PrEP-related care from a PCP

Over half (57.0%) of the GBM in this sample preferred receiving their PrEP-related care from a PCP. In bivariate analyses, we found significant differences in preference for receiving PrEP-related care from a PCP by age, race/ethnicity, income, relationship status, health insurance status, PCP provider access, access to an LGBT-friendly healthcare provider within 30 minutes travel time, and concern about bringing up the topic of PrEP with a doctor. All results of bivariate analyses can be found in *Table 1.* In the multivariable model examining preference for receiving PrEP-related care from a PCP, men of Latino or other/multiracial race/ethnicity had lower odds of preferring a PCP compared to White men. Although access to a PCP provider was not significantly associated with PCP preference for PrEP-related care, men with PCPs who were aware that they have sex with men had significantly higher odds of preferring to receive their PrEP-related care from a PCP compared to men who were not “out” to their PCP. Men with higher concern about quarterly medical check-ups every three months had significantly higher preference for receiving PrEP-related care from their PCP, but men with higher concern about bringing up the topic of PrEP with their doctor had significantly lower odds of preferring a PCP for PrEP-related care. No other variables were significantly associated with PCP preference for PrEP-related care in the multivariate model (see *Table 2*for complete results).

**Table 2:** Results of both fully-adjusted logistic regression models predicting 1) preference to receive PrEP-related care from a primary care provider and 2) interest in using home-based PrEP services (*n* = 906).

	Prefer Receiving PrEP-Related Care from a PCP^1^ (Ref: prefer specialist clinics and providers)			Prefer using Home-Based PrEP Services (Ref: not preferred)		
Predictor Variables	*AOR*	*95% CI*	*AOR*†	*AOR*	*95% CI*	*AOR*†
Age	1.00	0.98–1.01	0.95	0.98**	0.97–0.99	0.76
Race/Ethnicity (Ref: White)						
Black	0.79	0.45–1.40	0.94	0.96	0.53–1.75	0.99
Latino	0.56*	0.34–0.91	0.83	0.68	0.42–1.12	0.88
Other/Multiracial	0.55*	0.31–0.97	0.85	0.95	0.51–1.77	0.99
Education (Ref: Less than Bachelor’s degree)						
Bachelor’s degree or more	0.97	0.70–1.34	0.99	1.32	0.94–1.85	1.15
Employment (Ref: Unemployed)						
Employed (part-time or full-time)	0.86	0.54–1.39	0.95	1.34	0.84–2.13	1.11
Income (Ref: Less than $20k per year)						
$20k to $49k per year	1.00	0.61–1.62	1.00	0.96	0.57–1.60	0.98
$50k or more per year	1.33	0.79–2.23	1.15	1.18	0.69–2.01	1.08
Geographic Region (Ref: Northeast)						
Midwest	1.04	0.64–1.71	1.02	1.70*	1.02–2.85	1.23
South	0.99	0.64–1.53	0.99	1.18	0.77–1.83	1.08
West	0.93	0.59–1.48	0.97	1.41	0.88–2.24	1.16
Relationship Status (Ref: Single)						
In relationship	1.02	0.74–1.43	1.01	1.02	0.72–1.44	1.01
Main Partner HIV-Status (Ref: HIV-negative, unknown, or no main partner)						
HIV-positive	0.89	0.45–1.76	0.97	0.65	0.33–1.27	0.90
Engaged in Sexual HIV Transmission Risk^2^ (Ref: No)						
Yes	0.94	0.67–1.31	0.97	1.72**	1.19–2.50	1.29
Recent HIV Testing (Within last 6 months; Ref: No)						
Yes	0.89	0.65–1.23	0.95	0.85	0.61–1.20	0.93
PrEP Intentions	1.01	0.86–1.19	1.01	1.44***	1.21–1.71	1.46
Has Health Insurance Status (Ref: No)						
Yes	1.34	0.77–2.32	1.09	1.18	0.66–2.10	1.05
Primary Care Provider (PCP) Access (Ref: Not "out" to PCP)						
No PCP	0.75	0.46–1.23	0.88	1.18	0.67–2.09	1.08
"Out" to PCP	3.37***	2.17–5.25	1.82	0.75	0.46–1.23	0.87
Within 30 Minutes of LGBT-Friendly Provider (Ref: Yes)						
No or don't know	0.89	0.64–1.24	0.95	1.50*	1.05–2.14	1.22
Concern about Medical Checkups Every 3 Months	1.23*	1.05–1.45	1.25	1.35***	1.14–1.59	1.37
Concern about Bringing up the Topic of PrEP with Doctor	0.51***	0.42–0.61	0.52	1.20	0.98–1.47	1.20
**Model Statistics**						
*F-*test (*df*)	*F*(22, 883) = 234.1***			*F*(22, 883) = 114.4***		
Psuedo *R*^2^	0.19			0.11		

* *p* < 0.05.

** *p* < 0.01.

*** *p* < 0.001.

^1^ PCP = primary care provider.

^2^ Any condomless anal sex with an HIV-positive or unknown partner and/or any casual partner in past 3 months.

† Standardized *AOR* reported to improve comparability between continuous variables measured using different metrics, as the *AOR* is based on a one standard deviation unit change in the variable.

### Preference using HB-PrEP

Most (72.3%) GBM were interested in HB-PrEP. Bivariate analyses are similarly reported in *Table 1.* Briefly, we found significant differences in preference for using HB-PrEP by age, employment status, engagement in sexual HIV transmission risk, PCP provider access, access to an LGBT-friendly provider within 30 minutes travel time, PrEP intentions, and concerns about medical checkups every three months and bringing up the topic of PrEP with a doctor. In multivariable regression, older men had significantly lower odds of preferring HB-PrEP. Men in the Midwest had significantly higher odds of preferring HB-PrEP compared to men in the Northeast; 77.4% of men in the Midwest preferred HB-PrEP, but men in the Northeast had the lowest frequency of preferring HB-PrEP (66.9%). Men who had engaged in sexual HIV transmission risk had higher odds of preferring HB-PrEP compared to those who had not, and men with higher intentions to initiate PrEP had higher odds of preferring HB-PrEP. GBM who did not live within 30 minutes travel time to an LGBT-friendly provider had higher odds of preferring HB-PrEP compared to men without, as did men who had more concern about receiving medical check-ups every three months. Other variables tested in our multivariable model were not significantly associated with preference for HB-PrEP (see *Table 2*for complete results).

## Discussion

In our nationwide cohort of GBM who had not yet initiated PrEP, more than half said they would prefer to receive PrEP-related care from a PCP; however, nearly three-quarters of men preferred to receive PrEP persistence care via HB-PrEP services. As men who are interested in receiving HB-PrEP would still need to go to a healthcare provider for their first visit for PrEP prescription, these data are relevant for both PrEP uptake *and* persistence among GBM. PCPs need to be prepared to discuss and prescribe PrEP to GBM, but just under half of GBM prefer to receive this care from a specialist clinic or provider. In response to the providers’ perspectives, including the “purview paradox” [38] indicating confusion for the best place for patients to receive PrEP-related care, GBM in this sample indicated interest in care from both PCPs and specialists.

Despite mixed healthcare provider preferences across the whole sample, the largest determinant of PrEP-related care provider preference was whether the participant was “out” to their PCP. We found no differences between GBM who were not “out” to their PCP and those who did not have a PCP on provider preference; thus, having a PCP is only important for preference if that provider is aware that he engages in same-sex sexual behavior. This finding adds to an existing body of literature identifying the importance of healthcare providers’ ability to provide a non-stigmatized environment for discussing sexual behavior with competent care for GBM [39–42]. This is particularly important because men who worried more about the quarterly medical checkups currently recommended for PrEP would prefer to engage in the frequent care with a PCP, yet men who had more concern about bringing up the topic of PrEP with a doctor preferred to get PrEP-related care from a specialist clinic or provider. Healthcare providers need to be similarly prepared to provide PrEP-related care; 24% of PCPs had not heard of PrEP and only 17% had prescribed it in the 10 cities with the highest HIV prevalence between July 2014 and May 2015 [43], but PCPs desire additional training and resources to provide competent PrEP-related care [44]. From these findings, appropriate public health messaging interventions should be tailored for PrEP uptake by indicating different options for GBM to obtain PrEP based on their individual concerns.

Although we found no significant differences between most demographic variables and provider preferences for PrEP-related care, GBM who were of Latino or other/multiracial race/ethnicity had significantly lower odds of preferring PrEP-related care from a PCP compared to Whites. Black GBM did not significantly differ from White GBM in our multivariable analysis of provider preference; however, we did find that about 53% of Black GBM preferred a PCP compared to over 60% for White GBM. Thus, our lack of significance could be the result of the limited number of Black GBM in our sample. Nonetheless, these findings conform with our current knowledge about barriers specific to non-White GBM. Race-based medical mistrust, distrust in the pharmaceutical industry, and sexuality stigmas are all barriers to PrEP uptake for non-White GBM [13, 14], which could be a reason these men prefer a specialist clinic or provider that can better relate with them than a typical PCP. In bivariate analyses, we also found GBM with higher income, health insurance, older age, and being partnered more frequently preferring care from a PCP; however, these plausible predictors of health care access were not significant after accounting for PCP access in our multivariate model. We found a similar attenuating effect on the correlation of LGBT-friendly provider access observed in bivariate analyses after adjusting for PCP access in the multivariate analysis. It is plausible that men who are “out” to their PCP stated that they had access to an LGBT-friendly provider, assuming this provider was within 30 minutes travel time as stated in our question.

After initial PrEP prescription, we found that a majority of the GBM in this sample would prefer using HB-PrEP for PrEP persistence. Both younger men and those who had recently engaged in sexual HIV transmission risk preferred HB-PrEP to the general model of PrEP-care delivery (i.e., returning for quarterly medical care visits for prescription renewal and HIV/STI testing). This is perhaps the most important finding in our research, as younger GBM engaging in HIV transmission risk are the most vulnerable population to HIV [1]. Based on our HB-PrEP findings, it is plausible that younger GBM may also be more inclined to use other newer technologies for HIV prevention (e.g., HIV self-testing), but further research exploring these other technologies is needed with younger GBM because of the relatively older age of our sample; nonetheless, we had sufficient variability in age with enough younger men to examine the impact of age on our outcomes of interest in this study. Moreover, GBM with higher intentions to initiate PrEP preferred HB-PrEP more frequently compared to those with less intentions, indicating we might best reach individuals who want to start PrEP by offering HB-PrEP. The expansion of HB-PrEP services has the potential to increase PrEP uptake among younger GBM at highest-HIV risk and those who plan to initiate PrEP; thus, clinical guidelines for PrEP use should consider providing recommendations for the use of HB-PrEP.

Regarding geographical differences, we found men in the Midwest preferred HB-PrEP more compared to men in the Northeast. HB-PrEP could resonate most with people who live greater distances away from a major city–there are more of these men in the Midwest than Northeast. Despite differences by race/ethnicity in healthcare provider preferences, we found no differences by race/ethnicity in our analyses with HB-PrEP. Issues of medical and pharmaceutical mistrust and stigma could matter most for the initial uptake of PrEP, but have less impact on the preference of care for PrEP persistence among non-White GBM. However, we caution minimizing the negative implications of stigma–particularly HIV pill stigma–on PrEP use because HB-PrEP does not attempt to ameliorate stigma associated with others thinking the PrEP user is HIV-positive or shame associated with being a PrEP user; both have been identified in prior literature [37].

The implications of preference for HB-PrEP based on health care access and concerns about frequent medical checkups has similar relevance to PrEP persistence, in addition to our aforementioned discussion on uptake. GBM without access to an LGBT-friendly provider within 30 minutes travel time had higher preference for HB-PrEP, indicating HB-PrEP has the potential to reduce barriers for continuation of PrEP-related care for those without proximal access. Limited access to an LGBT-friendly provider could indicate that they reside in an area with higher structural stigma against same-sex behavior [45]. This barrier could be more pronounced among GBM who live in rural areas where access to these services may be limited, which is plausible based on prior research that found nearly all (98%) PrEP users resided in metropolitan areas [46]. Furthermore, GBM with more concern about the quarterly medical checkups associated with PrEP use had higher preference for HB-PrEP similarly; thus, HB-PrEP is a mechanism to reduce their perceived barriers to PrEP persistence.

### Limitations

The strengths of this study should be understood in light of its limitations. The predominately-White sample in this study is indicative of the HIV-negative population of GBM in the US; White men outnumber their Black and Latino counterparts [47], and pre-existing and ongoing racial disparities in HIV precluded a lot of men of color from joining our study because they were HIV-positive [34]. Further research with larger samples of non-White GBM are needed to more fully study these issues given their higher-HIV risk compared to non-White GBM [1]. Second, all data are subject to the limitations associated with self-reported data collection procedures, for example multiple testing effects and potential response bias, despite our efforts to reduce demand effects with self-administered surveying. Third, we assessed sexual HIV transmission risk based on any condomless anal sex with an HIV-positive or unknown partner and/or any casual partner in the past three months, but we do not have data on whether the HIV-positive partner had an undetectable viral load. Fourth, our description of HB-PrEP services described receiving prescription mailing and at-home HIV/STI testing after an initial in-person, clinic-based visit. HB-PrEP in practice might differ after clinical recommendations are made, such as HB-PrEP offered after an initial clinic-based appointment and a three-month follow-up for more comprehensive physiological testing (e.g., kidney function). Fifth, the low pseudo *R-*squared values, while not a true measure of model uncertainty, provides some initial indication of unexplained variance; further qualitative research is needed to identify additional factors that might influence preference for PrEP-related care provider type and HB-PrEP. Sixth, we excluded men currently or previously on PrEP because of theoretical differences compared to those who have not yet initiated PrEP. Further research is needed about PrEP-related care received by current PrEP users, in addition to research on current PrEP-users’ preferences for HB-PrEP. Finally, GBM enrolled in this cohort have participated in at-home HIV and bacterial STI testing as part of this study at baseline; thus, preferences for HB-PrEP might differ from those who have not yet engaged in these at-home testing behaviors.

### Conclusion

In this US-nationally representative sample of GBM, men had split preferences of where they would like to receive their PrEP-related care, with being “out” to a PCP the largest determinant of preferences. Meaningful barriers of concern about frequent medical checkups were associated with preferring a PCP for PrEP-related care, but men who perceived a barrier to bringing up the topic of PrEP with a doctor preferred a specialist clinic or provider more than a PCP. HB-PrEP was more appealing for younger men and those engaged in sexual HIV transmission risk, suggesting HB-PrEP could help reach GBM most vulnerable to HIV and most in need of PrEP. The expansion of HB-PrEP has potential to increase PrEP uptake and persistence among GBM, particularly for men with barriers to clinic-based care and higher intentions to initiate PrEP. Therefore, we recommend the implementation and evaluation of HB-PrEP to guide the development of clinical guidelines for its use.
